# Disproportionate Availability Between Emergency and Elective Hand Coverage: A National Trend?

**Published:** 2016-09-09

**Authors:** Stella Y. Chung, Aditya Sood, Mark S. Granick

**Affiliations:** Division of Plastic Surgery, Department of Surgery, Rutgers New Jersey Medical School, Newark

**Keywords:** emergency hand coverage, elective hand coverage, hand trauma, disproportionate availability, national trend

## Abstract

**Background**: Traumatic hand injuries represent approximately 20% of emergency department visits; yet, access to emergency care remains inadequate. Recent surveys from several states report a wider availability of hand specialists providing elective care than emergency care. The authors aim to examine this phenomenon in the state of New Jersey and whether there is a national trend toward disproportionate availability between emergency and elective hand coverage. **Methods**: A survey was conducted of all New Jersey hospitals, excepting university hospitals, in August 2014. To assess the availability of hand surgery coverage, the following questions were asked: (1) Does your hospital provide elective hand surgery? and (2) Is there a hand specialist/surgeon on call always, sometimes, or never? **Results**: A total of 58 hospitals were called, with a 67.2% response rate (*n* = 39). The majority (87.2%) of hospitals offered elective hand surgery, whereas only 64.1% provided immediate 24/7 hand coverage. Only 38.5% of hospitals located in the same county as a level I trauma center provided 24/7 emergency hand care, whereas 76.9% of hospitals in counties without any level I trauma center did (*P* < .05). Cities with a higher poverty level were less likely to provide emergency coverage than cities with a lower poverty level (47.4% vs 80.0%; *P* < .05). **Conclusions**: There is a discrepancy between emergency and elective hand care in New Jersey. Similar findings across the nation suggest a concerning trend of limited access to emergency hand health care. Alternative systems that can appropriately triage and treat patients are warranted.

Traumatic wrist, hand, and finger injuries represented approximately 9.8% of all visits to emergency departments (EDs) in 2010.^1^ Many published reports have cited inadequate access to emergency hand care in various regions of the United States.^2^^-^9 A study conducted in California reported that emergency hand consultations are among the most difficult to obtain in the ED.^2^ This deficiency is of concern since 21% of permanent injuries and disabilities arising from ED visits are caused by delayed specialty care due to inadequate availability of consultants.^10^ Recent surveys from California, north Florida, upstate New York, and Tennessee report a wider availability of hand specialists providing elective care than emergency care.^3^^-^6 This indicates that hand specialists are providing elective care without providing on-call services. We aim to determine the availability of emergency and elective hand coverage in New Jersey, the most densely populated state in the United States. In New Jersey, there are no remote areas with deficiencies of medical specialists.

## METHODS

A list of hospitals in the state of New Jersey and their locations was accessed from the New Jersey Hospital Association Web site (www.njha.com). A total of 58 hospitals with full-service EDs were included in the analysis after excluding main teaching institutions, as emergency hand care services are empirically provided by in-hospital residents. A survey of the medical directors or staff coordinators was conducted through telephone, e-mail, and fax. Medical directors or staff coordinators were chosen, because they are most likely to be knowledgeable of the hospital staff. The following questions were asked: (1) Does your hospital provide elective hand surgery? and (2) Is there a hand specialist/surgeon on call always, sometimes, or never? Nonresponders were called at least 2 more times at 1- to 3-week intervals after the initial contact. Additional data collected from the survey were the hospital name and respondent position. Data collection was completed in August 2014. Survey participants were informed that their responses will be recorded for future research, participation was voluntary, and all identifiers will be removed for confidentiality.

To determine the socioeconomic status of the cities in New Jersey, the percentage of persons below poverty level by city was accessed from US Census Bureau's 2006-2010 American Community Survey (http://factfinder.census.gov). Addresses of respondent hospital were obtained from the hospital Web sites. Literature regarding hand surgery coverage in other states was searched using key words related to emergency hand coverage on PubMed.

Collected data from the survey were saved into a database on a secure server in the medical office by the primary author. Incomplete data from nonresponders were designated as missing data, not as negative response. Statistical analysis was performed with Fisher exact tests for categorical variables with the software STATA (version 13.0).

## RESULTS

A total of 58 hospitals were called to determine the availability of emergency versus elective hand specialists. The response rate was 67.2% (39/58). Of the respondents, 87.2% (34/39) provided elective hand coverage, 64.1% (25/39) had 24/7 emergency hand coverage, and 17.9% (7/39) reported no emergency hand care at all. Of the hospitals that provided elective hand coverage, 70.6% (24/34) had 24/7 on-call availability.

**Figure 1:**
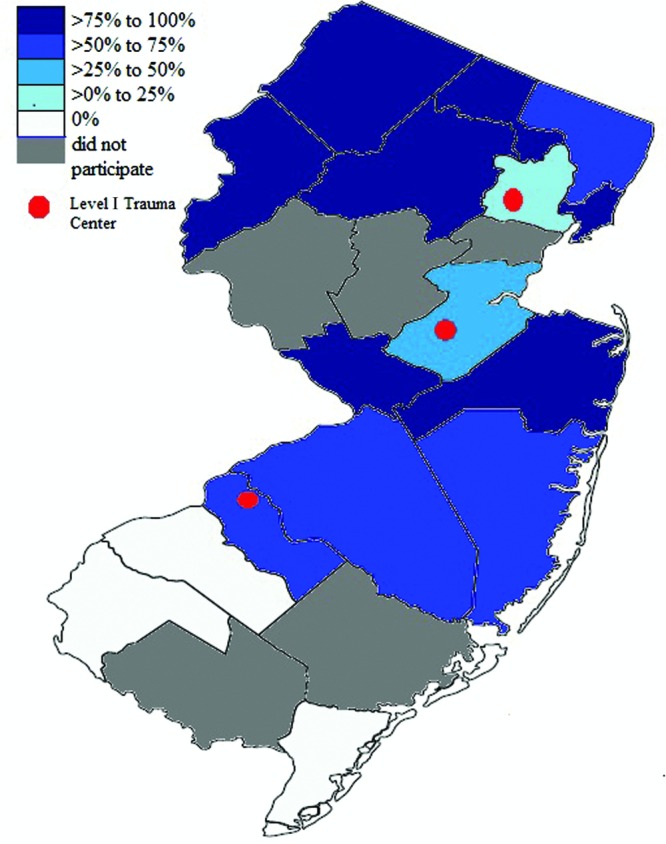
Provision of emergency hand coverage in relation to level I trauma center proximity. Three level I trauma centers in New Jersey are marked in red.

Subsequently, the effect of geographic location on the availability of emergency versus elective hand coverage was examined. There are 3 level I trauma centers in New Jersey (Fig 1). Hospitals within a county where a level I trauma center is located were significantly less likely to provide 24/7 hand coverage than hospitals in a county without a level I trauma center (38.5% vs 76.9%; *P* < .05). Although fewer hospitals offered elective hand care when there is a level I trauma center within the same county, this was not statically significant (84.6% vs 88.5%; *P* < .05).

**Figure 2:**
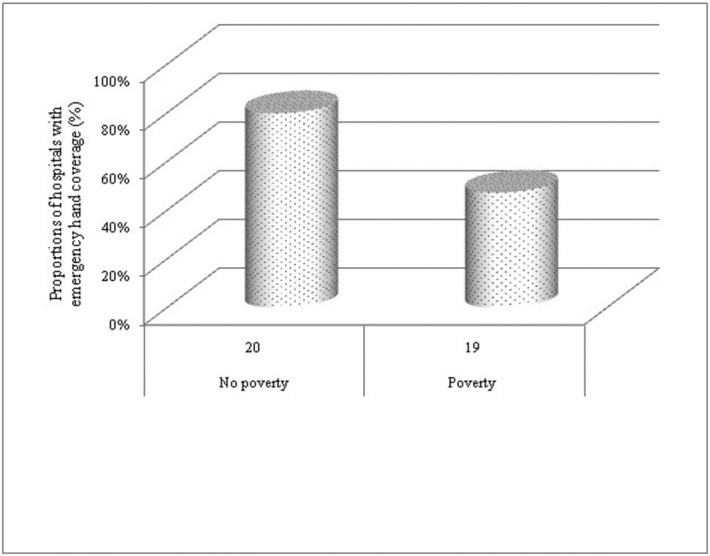
Proportions of hospitals that provide emergency hand coverage distributed by socioeconomic factors at the city level (%), *P* < .05. Less than state average is labeled as “no poverty,” and above state average is labeled as “poverty”; n represents total sample size. *P* value by the Fisher exact test.

Elective and emergency hand coverage was distributed by regional socioeconomic status and population demographics. Socioeconomic standing was measured by median household income and the percentage of persons below poverty level. Population demographic was determined by the percentage of nonwhites, which included African Americans, Hispanics, Native Americans, and Asians. The high poverty-level cities were significantly less likely to provide emergency coverage than cities with a lower poverty level (47.4% vs 80.0%; *P* < .05; Fig 2).

**Figure 3:**
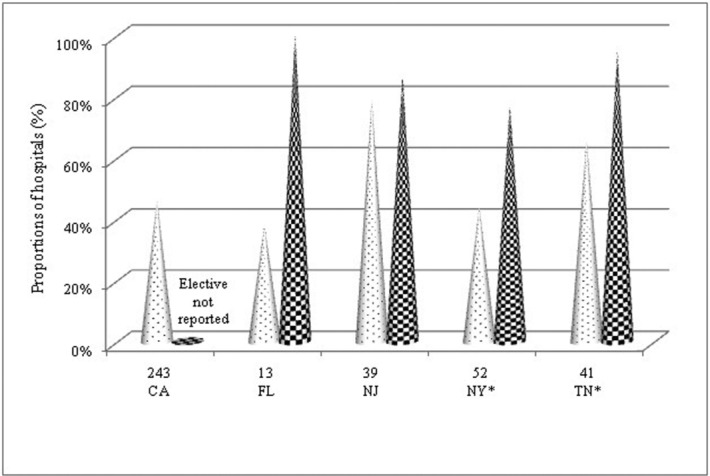
Availability of emergency and elective hand coverage in 5 US states (%). The proportion is based on survey respondents, *P < .05. Dotted represents emergency, checkered represents elective, and n represents total sample size. California indicates CA; FL, Florida; NJ, New Jersey; NY, New York; TN, Tennessee. *P* value by the Fisher exact test.

Accessibility of elective and emergency hand coverage in 5 states, including the present study, is compared. Elective coverage was higher in all states (Fig 3). Because of the differences in data analysis and reported outcomes, results could not be combined as a meta-analysis.

## DISCUSSION

This retrospective study of general hospitals in New Jersey shows a wide gap between hand surgeons available for emergency and elective hand services. Eighty-seven percent of the respondents provided elective coverage, whereas 64% provided 24/7 on-call care. Furthermore, approximately 30% of the hospitals that offer elective hand surgical procedures did not provide 24/7 emergency hand coverage. Such discrepancy in hand surgery is not a singular finding in New Jersey, but it also has been reported in California, Tennessee, upstate New York, and north Florida.^3^^-^6 This phenomenon raises a concern about the availability of hand surgeons in the United States for needed emergency care.

Davison^7^ in 2004, as well as Caffee and Rudnick^5^ in 2007, reported an evolving crisis with ED coverage by hand surgeons. Their concern was spurred on by the closings of EDs and downgrades of trauma centers due to the lack of physician coverage at the time.^7^^,^^10^ A growing number of plastic surgeons were dropping their ED privileges secondary to high malpractice insurance costs, government financial subsidies whose benefits excluded physicians, and decreasing reimbursement rates for ED coverage while EDs were becoming the source of primary care for the uninsured population. Nationally, only 50% of all emergency care is financially compensated.^8^^,^^9^ In a retrospective study of 300 patients for 30 months, the collection rate of plastic surgery ED visits was found to be as low as 13.96% of the total billed amount.^7^ The reimbursement rate for elective surgery was not reported. This extremely skewed risk-to-reward ratio has been influencing hand surgeons to practice outpatient elective care while removing themselves from emergency care,^5^^,^^7^ resulting in both hand and plastic surgery coverage being the most difficult surgical specialties to obtain in the ED.^2^

The Patient Protection and Affordable Care Act was signed into law in 2010. Year-to-year comparison is difficult due to the recent change in government survey methodology,^11^ but the uninsured population is expected to decrease. Physicians in New Jersey have the lowest participation in Medicaid in the United States.^12^ Not surprisingly, surveys show that disparity between emergency and elective hand coverage still exists despite 31% increase in demand for emergency care in the last decade.^3^^-^6^,^^13^ Shortage of on-call hand surgeons means delayed or lack of emergency care. Wait time is longer and patients are often transferred to other hospitals, typically level I trauma centers, regardless of the urgency of patient condition.^10^^,^^14^^,^^15^ In addition, improper or inadequate initial treatment often sets the course of recovery that results in complications and/or permanent disabilities.

We examined how geography influences the availability of hand surgeons. Hospitals within the vicinity of level I trauma centers offered significantly less emergency service than hospitals without level I trauma center within the county. A level I trauma center is a regional resource facility with the capability and equipment to provide total care for full range of trauma at all times. In New Jersey, there are 3 university-affiliated level I trauma centers that provide 24/7 hand coverage located in 3 counties.^16^ There is a high volume of transfers and referrals to these institutions, including ours, due to the lack of emergency coverage in the neighboring community hospitals. Nationally, more than half of all transfers to level I trauma centers are reported to be due to the lack of on-call specialist coverage.^17^^,^^18^ Among these referring hospitals are level II trauma centers, which are progressively becoming underequipped for large patient volume due to physician shortage.^17^^,^^18^ Consequently, more patients are admitted outside of their own county.^19^ As Emergency Medical Treatment and Active Labor Act (EMTALA) legally requires level I trauma centers to accept incoming transfers, the already crowded institutions must bear the additional strain on resources and financial burden.^20^^-^22 Plastic surgery is one of the specialties that is heavily impacted by this unfunded mandate.^2^ Another worrisome finding is the lack of hand specialists on call in select level I trauma centers in the United States despite the legal requirements for the surgeons to be available at all times.^4^ A lack of emergency hand coverage at community hospitals may be acceptable if hand surgeons are available at regional level II and III trauma centers. However, there is considerable stress on tertiary centers due to the overwhelming number of patients who could have been readily managed at community hospitals.

Regional socioeconomic status seems to influence the availability of on-call hand surgeons. Cities with higher poverty rate were significantly associated with gaps between emergency and elective care access. Similar finding has been reported from Tennessee.^4^ This correlation may explain the inadequate emergency coverage and high referral rates from the hospitals within the vicinity of level I trauma centers. Level I trauma centers in New Jersey are located in socioeconomically disadvantaged regions. The neighboring hospitals are located in the cities with an average poverty rate of 13.4%, which is greater than the state average of 9.10%.^23^ It is likely that financial disincentive for hand surgeons to offer emergency hand care is the main reason for lack of coverage in these areas. Studies report that low-income patients are often underinsured, more likely to experience interhospital transfers, and ultimately may suffer from disruptions in care.^7^^,^^24^^,^^25^ Referrals to tertiary centers should be reserved for complicated hand trauma cases and not based on patient's socioeconomic and/or insurance status.

Emergency hand coverage is essential. However, there is a low availability of emergency coverage despite sufficient manpower performing elective hand surgery. Consequently, alternative ways to address the lack of emergency coverage are necessary. National guidelines for initial evaluation must be developed to avoid inappropriate triage to tertiary health care facilities. Emergency physicians and physician extenders should be better educated in the evaluation and initial treatment of hand injuries. Hartzell et al^26^ reported that many of the hand injuries in the ED can be treated without specialty involvement. Another solution would be to deploy telemedicine, an effective tool that is not widely used for emergency evaluations. Telemedicine has been approved for chronic wound care in 21 states and has been implemented in some Veterans Healthcare systems, federally funded grants, closed medical systems such as Kaiser, Marshfield Clinic, prisons, military hospitals, individual hospitals, and wound centers.^27^ As health information technology and personal health information protection technology are rapidly improving, telemedicine has the capability to provide equal access to care, limit inappropriate transfers, improve efficiency and continuity of care, and facilitate information exchanges. In addition, government initiatives to provide appropriate resources for burdened trauma centers are needed.

Limitations of this study include the particular demographics of New Jersey and may not be representative of all parts in the United States, particularly the more rural regions. Nonresponder bias also must be considered; however, our response rate of 67% falls within the range of previously published regional surveys.^3^^-^6 Recall bias, reliability, and validity of the responses must also be considered. We tried to minimize recall bias by corresponding with the director of ED or person in a managerial position who is more likely to be knowledgeable of the daily operation of his or her hospital staff.

Hand surgery trauma call encompasses a wide spectrum of responsibility and is considered in conjunction with the emergency responsibilities of orthopedic surgery, plastic surgery, and hand fellowship–trained surgery services.^28^ To our knowledge, this is the first study that examined the availability of New Jersey emergency hand care. Our statewide survey of hospitals with full-service EDs showed a discrepancy between emergency and elective hand care in New Jersey. Similar findings across California, north Florida, upstate New York, and Tennessee suggest a disconcerting national trend of limited access to emergency hand health care compared with elective coverage.^3^^-^6 We also found that vicinity to a level I trauma center and a high poverty rate are important factors in predicting availability of emergency hand coverage. Disproportionate availability between emergency and elective care may lead to suboptimal patient care, patient “dumping” to tertiary care centers, and inappropriate utilization of resources. Several solutions to provide proper and efficient emergency hand care are proposed. They include reallocation of resources, coordinated distribution of care at a statewide level, and alternative approaches.

## CONCLUSIONS

Emergency department coverage has historically been the cornerstone upon which new hand surgeons built their practice.^7^ This tradition seems to be fading, as hand surgeons are less involved in emergency than elective care, particularly in regions within the vicinity of a trauma I level center and with a high poverty rate. It is of concern that many hospitals offer elective hand surgery but little to no emergency coverage. A national discussion is warranted to resolve this growing problem.
